# Subglottic giant adenoid cystic carcinoma: a case report

**DOI:** 10.3389/fsurg.2026.1687106

**Published:** 2026-03-12

**Authors:** Chenguang Zhang, Chenghao Hu, Yicong Wang, Jiping Zhao, Chaohua Wang, Bin Guo

**Affiliations:** 1 School of Clinical Medicine, Qinghai University, Xining, Qinghai, China; 2 Department of Otolaryngology, Affiliated Hospital of Qinghai University, Xining, Qinghai, China; 3 Department of Gastrointestinal Surgery, The Third Affiliated Hospital of Sun Yat-sen University, Guangzhou, China; 4 Pathology Department, Affiliated Hospital of Qinghai University, Xining, Qinghai, China

**Keywords:** adenoid cystic carcinoma, case report, ethics, radiotherapy, subglottic disease diagnosis

## Abstract

**Background:**

Adenoid cystic carcinoma (ACC) arising in the subglottic larynx is extremely uncommon. Because of its slow but locally invasive and neurotropic growth, diagnosis is often delayed until the tumor becomes advanced. Reporting such cases is valuable for raising clinical awareness and guiding management.

**Case description:**

We describe a 72-year-old woman with a 6-year history of cough and progressive shortness of breath accompanied by intermittent stridor, initially misdiagnosed as chronic pulmonary disease. Her comorbidities included grade-3 hypertension, pulmonary hypertension, fatty liver disease, gallstones, coronary atherosclerosis, pleural thickening, and a left diaphragmatic hernia. Flexible fiberoptic laryngoscopy showed a large pedunculated mass with its base in the subglottic region, prolapsing into and out of the glottis during respiration and nearly obstructing the airway. The airway was secured with an emergency tracheostomy, followed by transoral endoscopic removal using plasma radiofrequency under suspension laryngoscopy. Histopathology confirmed ACC with cribriform and solid patterns (grade II). The patient declined further surgery and radiotherapy; despite repeated contact attempts, no long-term follow-up information was available.

**Conclusion:**

Subglottic ACC can mimic lower-airway disease by causing dynamic glottic obstruction. Early laryngoscopic evaluation should be considered in patients with persistent dyspnea unresponsive to conventional treatment. Individualized airway management and, when possible, definitive oncologic therapy are key to improving patient outcomes.

## Introduction

Adenoid cystic carcinoma (ACC) is a rare, low-grade malignant tumor of glandular origin. It most often arises in the salivary glands of the head and neck, but may also occur in the airway and lacrimal glands, while involvement of the larynx is extremely uncommon (1, 2). Laryngeal malignancies are predominantly squamous cell carcinoma (SCC), accounting for 85%–95% of cases (3), whereas ACC contributes to <1% of all laryngeal cancers (4, 5). According to published data, fewer than 100 cases of subglottic ACC have been reported worldwide (6), with only occasional reports from China in recent decades (7). The incidence is higher in females (8, 9). Histologically, ACC is classified into cribriform, tubular, and solid subtypes (10); the cribriform pattern is most common and generally associated with a better prognosis, while the solid subtype is less frequent but more aggressive (11). Although ACC typically shows indolent growth, it has a strong tendency for local invasion, recurrence, and distant metastasis (12). Here, we present a rare case of primary subglottic ACC with both cribriform and solid components confirmed by histopathology, and review the literature to highlight its clinical features, diagnostic challenges, and management.

## Case presentation

**Figure 1:**
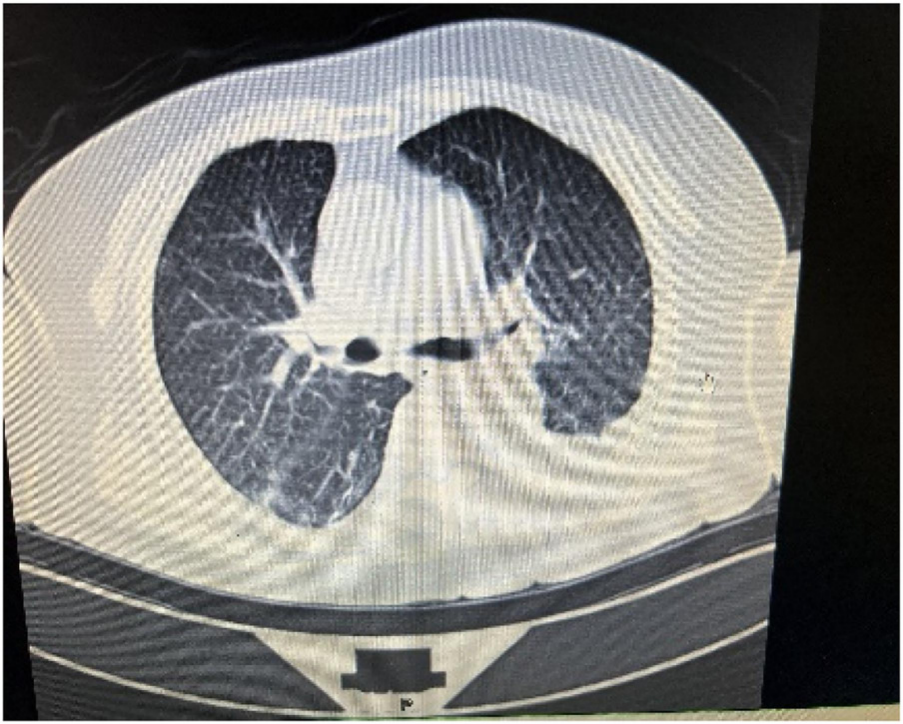
Chest CT on admission. Extensive inflammatory changes were observed in both lungs, accompanied by a marked left-sided pleural effusion.

**Figure 2:**
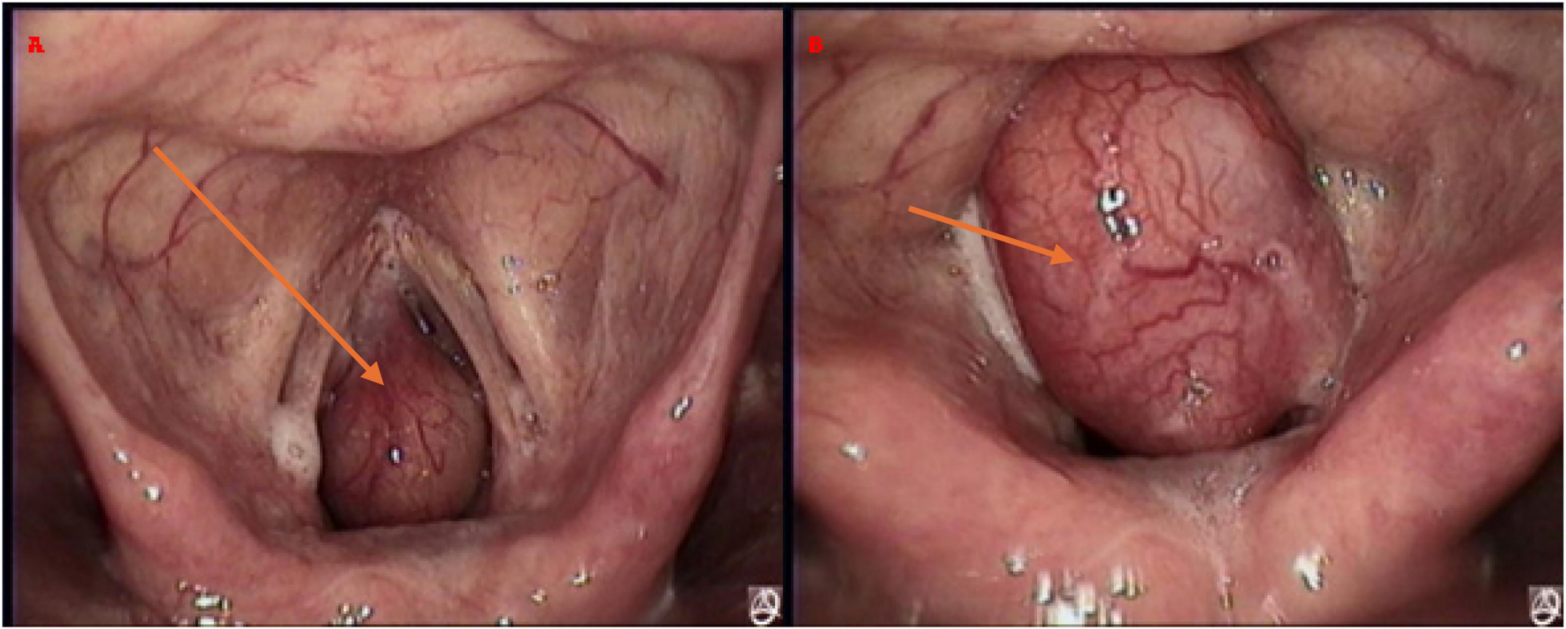
Electronic laryngoscopic images of the subglottic tumor. **(A)** During inspiration, the tumor is located below the glottis. **(B)** During expiration, the tumor is displaced above the glottis.

**Figure 3:**
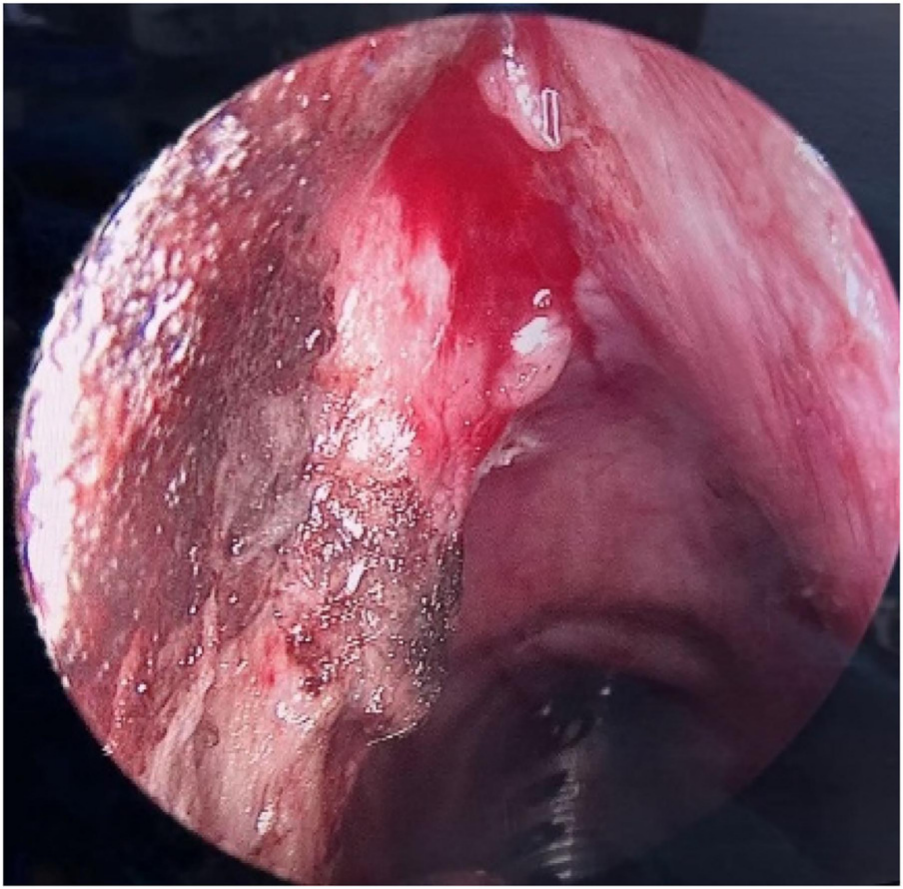
Intraoperative findings under suspension laryngoscopy. After plasma knife resection of the tumor, the intraoperative wound surface showed congestion, edema, and mild exudation.

**Figure 4:**
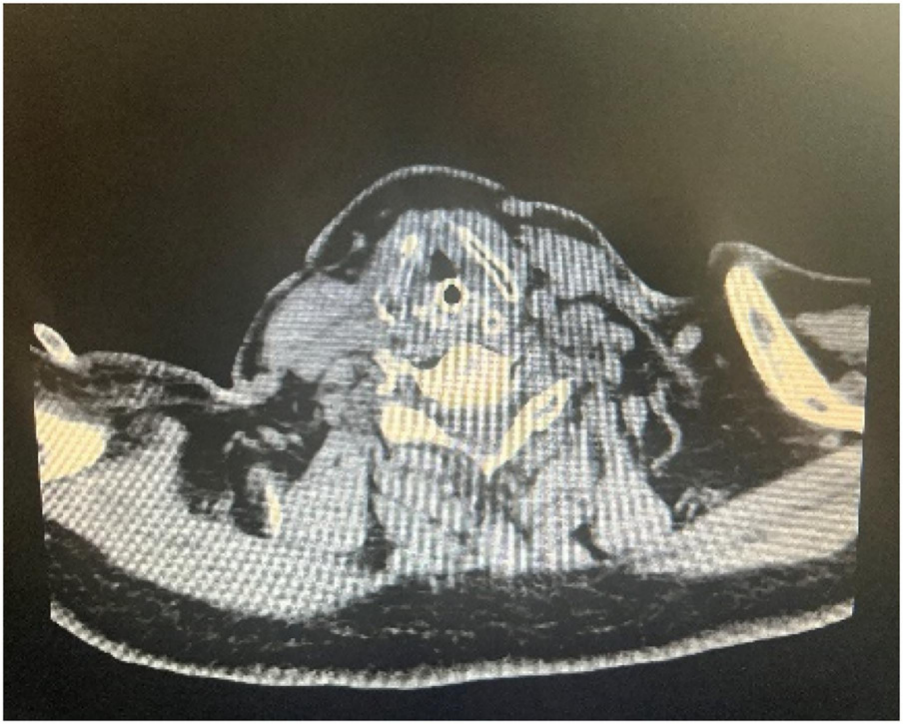
Postoperative cervical CT findings. No obvious stenosis or obstruction was observed in the glottic region, and the tracheal morphology was normal.

A 72-year-old woman presented with a 6-year history of intermittent cough, sputum, chest tightness, and dyspnea. One week before admission, her symptoms worsened after a common cold. At a local hospital, chest CT suggested chronic bronchitis, right lung atelectasis with suspected lobar syndrome, mucus plugging in the main bronchi, aortic and coronary atherosclerosis, mediastinal and hilar lymph node calcification, thickening of the left interlobar pleura, and diaphragmatic hernia. Laboratory tests showed leukocytosis (10.27 × 10⁹/L; neutrophils 76.1%, lymphocytes 17.9%) and elevated C-reactive protein (25.16 mg/L; high-sensitivity CRP >10 mg/L). She was diagnosed with chronic obstructive pulmonary disease with acute exacerbation (COPD-AE), pulmonary atelectasis, pulmonary hypertension, and grade-3 hypertension, and received cefoperazone–sulbactam, ambroxol hydrochloride, and doxofylline. Although cough and sputum improved slightly, chest tightness, dyspnea, and inspiratory stridor progressed, and she developed marked inspiratory distress and dysphagia even at rest. On examination, suprasternal and supraclavicular retractions and intercostal indrawing were observed. Because symptoms persisted despite standard COPD treatment, upper airway obstruction was suspected, and she was referred to our hospital. Chest CT (Figure 1) revealed right middle-lobe consolidation and atelectasis with intralesional calcification, left pleural effusion, and interstitial changes in both lungs. Flexible fiberoptic laryngoscopy identified a broad-based, pedunculated mass arising from the left subglottic region, prolapsing into and out of the glottis with respiration and nearly occluding the inlet (Figure 2). The surface was smooth and vascularized; left vocal cord mobility was preserved, but glottic closure was incomplete. An urgent otolaryngology consultation was obtained. Because of grade III laryngeal obstruction, the patient underwent emergency tracheostomy under general anesthesia followed by transoral endoscopic excision using plasma radiofrequency under suspension laryngoscopy. The mass was removed in a gross-total, piecemeal fashion; intraoperatively, it appeared smooth, firm, and well demarcated (Figure 3). No intraoperative frozen section was performed, and margin status was indeterminate due to piecemeal excision. After surgery, dyspnea and chest tightness improved significantly. She was treated with budesonide plus acetylcysteine nebulization and intravenous cefuroxime, which relieved cough and sputum production. A follow-up cervical CT prior to discharge showed bilateral thyroid atrophy with decreased density, multiple small lymph nodes in the carotid sheaths, and tortuous vessels around the left external carotid artery (Figure 4).

Two weeks after surgery, follow-up laryngoscopy showed pseudomembrane formation in the left glottic and subglottic regions, without evidence of significant stenosis, consistent with postoperative changes. Based on the pathological findings, partial laryngectomy followed by postoperative radiotherapy (PORT) was advised. However, because of the patient's age (72 years), multiple comorbidities, and concern about treatment risks, she and her family declined further therapy.

## Pathological diagnosis

**Figure 5:**
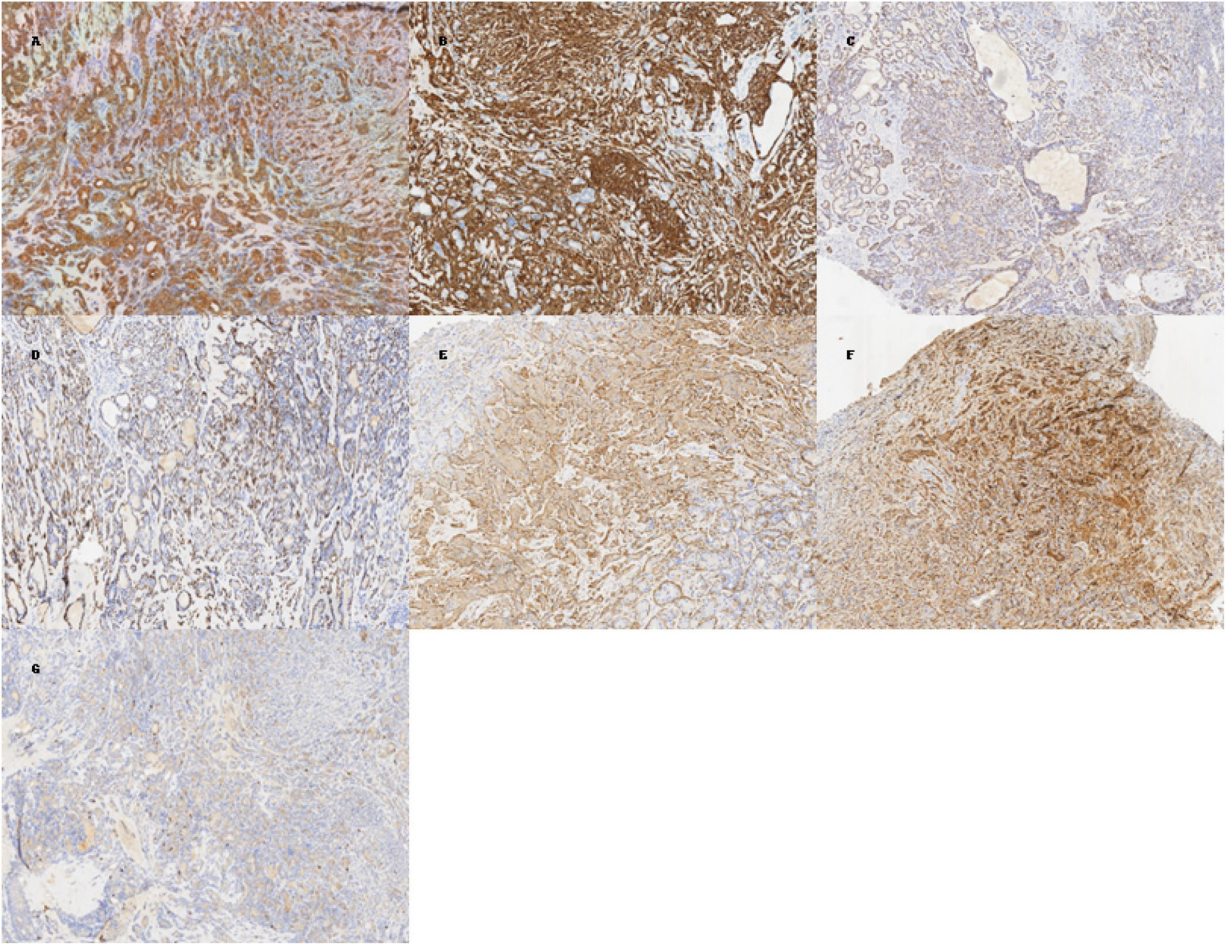
Pathological examination. **(A)** CK7 (+) **(B)** CK5/6 (+) **(C)** P63 (+) **(D)** P40 (+) **(E)** SMA (Myoepithelial +) **(F)** S100 (+) **(G)** Ki67 (5%–10%).

Grossly, the resected specimen consisted of multiple gray-white to gray-brown tissue fragments, measuring about 2.5 × 2 × 1.3 cm in total. Microscopically, the tumor showed features consistent with ACC, composed of both cribriform and solid patterns (grade II). A dual-cell population was identified: luminal ductal cells formed pseudocystic and cribriform structures, while abluminal myoepithelial cells surrounded these nests. Immunohistochemistry further supported the diagnosis: luminal cells were positive for CK7, CK8/18, and CD117 (partial), whereas myoepithelial cells were positive for p63, p40, calponin, SMA, S100, and CK5/6. The Ki-67 proliferation index was approximately 5%–10%, consistent with the low-to-moderate proliferative activity typical of ACC. Taken together, these morphologic and immunophenotypic features confirmed the diagnosis of ACC with cribriform and solid components (Figure 5).

## Discussion

Laryngeal malignancies rank second among respiratory system tumors and are common head and neck cancers, with SCC being the predominant type and men more frequently affected than women. ACC of the larynx is rare, and its occurrence in the subglottic region is exceedingly uncommon (3). ACC is characterized by slow growth, aggressive local invasion, a strong tendency for perineural spread, high recurrence rates, and a propensity for distant metastasis. The clinical signs and symptoms of laryngeal ACC depend on tumor size and location: supraglottic tumors typically present with dysphagia, glottic involvement with hoarseness, and subglottic tumors with stridor, dyspnea, and airway obstruction (4, 7). In the present case, the tumor originated in the subglottic region, where a large mass obstructed the glottis and moved with respiration. Thus, the patient primarily presented with stridor and dyspnea rather than hoarseness. Therefore, clinical assessment of ACC should integrate both tumor growth patterns and anatomical location.

The pathogenesis of ACC remains incompletely understood. The most frequently described molecular alteration is the chromosomal translocation t(6;9)(q22-23;p23-24), which produces the MYB–NFIB fusion gene and is detected in over half of cases (13). Aberrant expression of several microRNAs has also been implicated, influencing tumor growth, invasion, and metastasis (14, 15). In addition, dysregulation of signaling pathways such as Notch and PI3K/AKT/mTOR has been reported, contributing to cell survival and proliferation (13). Unlike squamous cell carcinoma, no clear link between smoking and ACC has been established, although smoking-related conditions such as pulmonary Langerhans cell histiocytosis have been associated with solid tumors, suggesting a possible indirect relationship that merits further study (16).

Clinically, ACC is an indolent but highly malignant tumor. In the larynx, its surface is often covered by normal-appearing mucosa, leading to long asymptomatic intervals and frequent misdiagnosis or late diagnosis. In some cases, delayed recognition may be fatal (8). One report described a patient repeatedly misdiagnosed with bronchial asthma, receiving standard therapy without improvement until acute airway obstruction (>90%) revealed the true diagnosis (3). Similarly, our patient, repeatedly diagnosed with chronic pulmonary disease due to recurrent cough, chest tightness, and dyspnea, only received an ACC diagnosis when tracheostomy was required to prevent asphyxia. Limited diagnostic thinking and delayed use of fiberoptic laryngoscopy contributed to the delay. Differential diagnosis is critical for subglottic ACC because of its overlap with other laryngeal diseases: (1) Laryngeal SCC: histologically composed of keratinizing or non-keratinizing squamous cells with carcinoma nests and necrosis. Unlike SCC, ACC tends toward perineural invasion and distant metastasis rather than lymphatic spread. (2) Rosai-Dorfman disease (RDD): a rare benign histiocytic proliferation with cervical lymphadenopathy; immunohistochemistry (S100 and CD68 positivity) distinguishes it from ACC (17). (3) Laryngeal papilloma: HPV-related, with papillary fibrovascular cores covered by squamous epithelium; lacks invasive or perineural features. (4) Subglottic stenosis: often secondary to trauma or intubation; CT/MRI reveals localized fibrosis with clear margins but no mass formation.

The optimal treatment for laryngeal ACC remains controversial. For subglottic tumors, total laryngectomy is generally considered the standard of care, though partial laryngectomy may be feasible in selected cases (4, 18). PORT may be considered to reduce recurrence risk (19). Conventional photon/electron radiotherapy and chemotherapy are mainly used in unresectable or metastatic cases. More recently, carbon ion radiotherapy has shown promising efficacy in selected patients. The prognostic role of histological grading remains debated, but given the slow natural course of ACC, follow-up should extend to at least 10 years (20). In the present case, the patient required emergency tumor debulking and tracheostomy to relieve life-threatening obstruction. Tracheostomy remains effective for rapidly alleviating airway compromise. Thus, clinical management should be tailored to the patient's condition, balancing tumor control with overall health status. In elderly patients with multiple comorbidities, the decision between aggressive oncologic treatment and palliative or supportive care requires careful ethical consideration. In this case, after thorough counseling regarding the potential benefits and risks of further surgery and radiotherapy, the patient declined additional treatment, reflecting a patient-centered balance between survival benefit and quality of life.

This case also highlights the importance of multidisciplinary collaboration. Fiberoptic laryngoscopy by ENT specialists led to tumor detection, and definitive diagnosis relied on pathology and immunohistochemistry. Given ACC's neurotropic nature and high recurrence risk, long-term surveillance is mandatory. Clinicians should maintain suspicion for rare diseases in patients with unexplained cough, dyspnea, or refractory symptoms, ensuring timely referral, endoscopic evaluation, and pathological confirmation. Ultimately, individualized treatment and prolonged follow-up are essential for optimal patient outcomes.

## Data Availability

The original contributions presented in the study are included in the article/Supplementary Material, further inquiries can be directed to the corresponding author.
